# Supplementary material for: Immunogenicity of Intradermal Versus Intramuscular BNT162b2 COVID-19 Booster Vaccine in Patients with Immune-Mediated Dermatologic Diseases: A Non-Inferiority Randomized Controlled Trial
Source: Vaccines (Basel). 2024 Jan 11;12(1):73. doi: 10.3390/vaccines12010073 (PMC10819288; doi:10.3390/vaccines12010073)
Supplement: Supplementary file 1 [file vaccines-12-00073-s001.zip › Supp1_Details of adverse event monitoring revision 2 09012024.pdf]

Supplementary materials for Immunogenicity of Intradermal Versus Intramuscular BNT162b2 COVID-19 Booster Vaccine in Patients with Immune-Mediated Dermatologic Diseases: A Non-Inferiority Randomized Controlled Trial

Chutima Seree-aphinan[1,2], Ploysyne Rattanakaemakorn[1], Poonkiat Suchonwanit[1], Kunlawat Thadanipon[1,3], Yanisa Ratanapokasatit[1], Tanat Yongpisarn[1], Kumthorn Malathum[4], Pornchai Simaroj[5], Chavachol Setthaudom[6], Onchuma Lohjai[6], Somsak Tanrattanakorn[1] and Kumutnart Chanprapaph[1],* †

[1] Department of Medicine, Division of Dermatology, Faculty of Medicine Ramathibodi Hospital, Mahidol University, Bangkok, Thailand, 10400 chutima.se@psu.ac.th (C.S.-a.); ploysyne.rat@mahidol.ac.th (P.R.); poonkiat.suc@mahidol.ac.th (P.S.); kunlawat.tha@mahidol.edu (K.T.); yanisa.rat@mahidol.ac.th (Y.R.); tanat.yog@mahidol.ac.th (T.Y.); somsak.tan@mahidol.ac.th (S.T.)

[2] Department of Internal Medicine, Division of Dermatology, Faculty of Medicine, Prince of Songkla University, Songkhla, Thailand, 90110

[3] Department of Clinical Epidemiology and Biostatistics, Faculty of Medicine Ramathibodi Hospital, Mahidol University, Bangkok, Thailand, 10400

[4] Department of Medicine, Division of Infectious Diseases, Faculty of Medicine Ramathibodi Hospital, Mahidol University, Bangkok, Thailand; kumthorn.mal@mahidol.ac.th, 10400

[5] Department of Ophthalmology, Faculty of Medicine Ramathibodi Hospital, Mahidol University, Bangkok, Thailand; pornchai.sim@mahidol.edu, 10400

[6] Immunology Laboratory, Department of Pathology, Faculty of Medicine Ramathibodi Hospital, Mahidol University, Bangkok, Thailand, 10400 chavachol.set@mahidol.ac.th (C.S.); onchuma.loh@mahidol.ac.th (O.L.)

* Correspondence: kumutnartp@hotmail.com, kumutnart.prb@mahidol.ac.th

† on behalf of COVIDVAC-DERM Study Group

Supplement 1: Details of adverse event monitoring during the study

The investigators will take the participants’ vital signs before and 30 minutes after the vaccine administration. The adverse reactions will be evaluated at 30 minutes after vaccination, then weekly for one month, monthly for three months, and six months after vaccination. The follow-up visits will take place in-person at the trial site except for week 2, 3, and 8 in which the participants were allowed to choose either in-person clinic visits or telemedicine visits.

<u>Vaccine-related adverse reactions</u>

Solicited adverse reactions will be documented using the relevant items listed in WHO’s Adverse Events Following Immunization (AEFI) form. The following AEFI items will be recorded.

- Local reactions:
  - Acute immunization site pain (pain that develops in response to vaccine administration): graded using Numerical Rating Scale (0-10)
  - Delayed immunization site pain (pain that develops in the minutes to hours following vaccination): graded using Numerical Rating Scale (0-10)
  - Itching
  - Induration
  - Nodule at injection site
  - Abscess
  - Cellulitis
  - Swelling of limb
  - Bleeding at injection site
  - Ipsilateral lymph node enlargement or lymphadenitis
  - Local reaction persisted > 3 days
  - Local reaction extended beyond the nearest joint
- Systemic:
  - Fever: < 38ºC, ≥ 38ºC
  - Headache
  - Chills
  - Arthritis
  - Generalised muscle pain
  - Fatigue or tiredness
  - Drowsiness
  - Fainting
  - Dizziness
  - Respiratory symptoms: cough, sore throat, nasal congestion
  - Gastrointestinal symptoms: abdominal pain, diarrhoea, loss of appetite, robust intake, nausea,

vomiting

- Neurological conditions: Hypotonic hyporesponsive episode (HHE), encephalitis, encephalopathy, seizures, aseptic meningitis, Guillain-Barre syndrome, narcolepsy
- Cutaneous reactions: urticaria, angioedema, urticarial vasculitis, leukocytoclastic vasculitis, morbilliform eruption, herpes zoster, chilblains, pityriasis rosea, Severe cutaneous adverse reactions (SCARs), others
- Anaphylaxis
- Thrombocytopenia
- Toxic shock syndrome
- Sepsis

<u>Disease activity, flare-ups, and treatment adjustment post-vaccination</u>

The following list of disease-related parameters will be recorded during each visit

- Koebner's phenomenon on the injection site
- Disease activity score:
    - For AIBD patients: Pemphigus Disease Area Index (PDAI), Bullous Pemphigoid Disease Area Index (BPDAI), Autoimmune Bullous Skin Disorder Intensity Score (ABSIS)
    - For psoriasis patients: Psoriasis Area Severity Index (PASI)
- The diagnosis of flare-ups: using separate case definition of each disease group
    - AIBD patients: the appearance of 3 or more new lesions (blisters, eczematous lesions, or urticarial plaques) or at least one large lesion (>10 cm diameter) a month that do not heal spontaneously within 1 week, or by the extension of established lesions, in a patient who has achieved disease control.
    - For psoriasis: Worsening of cutaneous disease activity leading to the initiation of new systemic immunosuppressive therapy or whole-body phototherapy.
- Treatment adjustment post-vaccination: using the case definition below
    - Down-titration of treatment: decrease the dose or discontinue the pre-existing systemic immunosuppressive therapies.
    - Up-titration of treatment: increase the dose of the pre-existing or initiate new systemic immunosuppressive therapies or whole-body phototherapy (for psoriasis).
    - Unadjusted: no adjustment of systemic immunosuppressive therapies up to the time of assessment.
