# Supplementary material for: Immunogenicity of Intradermal Versus Intramuscular BNT162b2 COVID-19 Booster Vaccine in Patients with Immune-Mediated Dermatologic Diseases: A Non-Inferiority Randomized Controlled Trial
Source: Vaccines (Basel). 2024 Jan 11;12(1):73. doi: 10.3390/vaccines12010073 (PMC10819288; doi:10.3390/vaccines12010073)
Supplement: Supplementary file 1 [file vaccines-12-00073-s001.zip › Supp2_supplementary tables and figures revision 2 09012024.pdf]

Supplementary materials for Immunogenicity of Intradermal Versus Intramuscular BNT162b2 COVID-19 Booster Vaccine in Patients with Immune-Mediated Dermatologic Diseases: A Non-Inferiority Randomized Controlled Trial

Chutima Seree-aphinan[1,2], Ploysyne Rattanakaemakorn[1], Poonkiat Suchonwanit[1], Kunlawat Thadanipon[1,3], Yanisa Ratanapokasatit[1], Tanat Yongpisarn[1], Kumthorn Malathum[4], Pornchai Simaroj[5], Chavachol Setthaudom[6], Onchuma Lohjai[6], Somsak Tanrattanakorn[1] and Kumutnart Chanprapaph[1],* †

1 Department of Medicine, Division of Dermatology, Faculty of Medicine Ramathibodi Hospital, Mahidol University, Bangkok, Thailand, 10400 chutima.se@psu.ac.th (C.S.-a.); ploysyne.rat@mahidol.ac.th (P.R.); poonkiat.suc@mahidol.ac.th (P.S.); kunlawat.tha@mahidol.edu (K.T.); yanisa.rat@mahidol.ac.th (Y.R.); tanat.yog@mahidol.ac.th (T.Y.); somsak.tan@mahidol.ac.th (S.T.)
2 Department of Internal Medicine, Division of Dermatology, Faculty of Medicine, Prince of Songkla University, Songkhla, Thailand, 90110
3 Department of Clinical Epidemiology and Biostatistics, Faculty of Medicine Ramathibodi Hospital, Mahidol University, Bangkok, Thailand, 10400
4 Department of Medicine, Division of Infectious Diseases, Faculty of Medicine Ramathibodi Hospital, Mahidol University, Bangkok, Thailand; kumthorn.mal@mahidol.ac.th, 10400
5 Department of Ophthalmology, Faculty of Medicine Ramathibodi Hospital, Mahidol University, Bangkok, Thailand; pornchai.sim@mahidol.edu, 10400
6 Immunology Laboratory, Department of Pathology, Faculty of Medicine Ramathibodi Hospital, Mahidol University, Bangkok, Thailand, 10400 chavachol.set@mahidol.ac.th (C.S.); onchuma.loh@mahidol.ac.th (O.L.)
* Correspondence: kumutnartp@hotmail.com, kumutnart.prb@mahidol.ac.th
† on behalf of COVIDVAC-DERM Study Group

Supplement 2: Supplementary Tables and Figures

Table S1. Clinical characteristics of the study participants grouped by assay sensitivity evaluation result.

	Immunogenicity data fulfil assay sensitivity assumption		p
	Yes (n=60)	No (n=48)	
Allocated to fID arm, n (%)	30 (50.0)	23 (47.9)	0.830[a]
**Age group, n (%)**			0.282[a]
< 65	41 (68.3)	28 (58.3)	
≥ 65	19 (31.7)	20 (41.7)	
**Female, n (%)**	35 (58.3)	24 (50.0)	0.387[a]
**Immune-mediated dermatologic disease, n (%)**			0.082[a]
Autoimmune bullous disease	30 (50.0)	32 (66.7)	
Psoriasis	30 (50.0)	16 (33.3)	
**Systemic immunosuppressants used before intervention, n (%)**			
Prednisolone, n (%)	16 (26.7)	20 (41.7)	0.100[a]
- Dose (mg/day), median (IQR)	5.6 (5.0-12.5)	5.0 (3.1-8.8)	0.161[b]
Azathioprine, n (%)	19 (31.7)	22 (45.8)	0.132[a]
- Dose (mg/day), median (IQR)	50.0 (25.0-100.0)	62.5 (25.0-75.0)	0.842[b]
Methotrexate, n (%)	15 (25.0)	10 (20.8)	0.610[a]
- Dose (mg/week), median (IQR)	10.0 (5.0-15.0)	12.5 (10.0-15.0)	0.535[b]
Mycophenolate mofetil, n (%)	2 (3.3)	1 (2.1)	0.694[a]
- Dose (mg/day), median (IQR)	2000.0 (1000.0-3000.0)	1000.0 (1000.0-1000.0)	0.480[a]
Cyclophosphamide, n (%)	1 (1.7)	0	0.369[a]
- Dose (mg/day), median (IQR)	14.3 (14.3-14.3)	0	NA
Ciclosporin, n (%)	4 (6.7)	1 (2.1)	0.260[a]
- Dose (mg/day), median (IQR)	100.0 (75.0-125.0)	50.0 (50.0-50.0)	0.263[b]
Sulfasalazine n (%)	5 (8.3)	1 (2.1)	0.159[a]
- Dose (mg/day), median (IQR)	3000.0 (2000.0-3000.0)	2000.0 (2000.0-2000.0)	0.527[b]
Leflunomide, n (%)	1 (1.7)	2 (4.2)	0.432[a]
- Dose (mg/day), median (IQR)	20.0 (20.0-20.0)	20.0 (20.0-20.0)	1.000[b]
Recent rituximab use[c], n (%)	4 (6.7)	2 (4.2)	0.573[a]
Interleukin 17/interleukin 23 inhibitors[d], n (%)	15 (25.0)	3 (6.3)	**0.009[a*]**
Tumour necrotic factor inhibitors[d], n (%)	1 (2.1)	0 (0)	0.261[a]
No systemic immunosuppressants used, n (%)	11 (18.3)	7 (14.6)	0.603[a]
**Interval between third and fourth dose (days), median (IQR)**	151.0 (128.5-183.0)	152.0 (136.0-180.5)	0.980[b]
**Previous COVID-19 vaccination, n (%)**			
Primary series			0.373[a]
- Viral vector vaccines	42 (70.0)	36 (75.0)	
- Inactivated vaccines	13 (21.7)	5 (10.4)	
- Heterologous vaccines	4 (6.7)	6 (12.5)	
- mRNA vaccines	1 (1.6)	1 (2.1)	
Third dose			0.260[a]
- mRNA vaccines	56 (93.3)	4 (97.9)	
- Viral vector vaccines	4 (6.7)	1 (2.1)	
**Baseline SARS-CoV-2-specific immunity levels, median (IQR)**			
Anti-SARS-CoV-2 S1 RBD IgG (bau/ml)	263.3 (121.2-691.8)	549.5 (258.6-1817.4)	**0.023[b*]**
- % participants tested negative (< 7.1 bau/ml)	2 (3.3)	1 (2.1)	0.694[a]
IFN-γ measured from SARS-CoV-2 IGRA (mIU/ml)	662.5 (217.9-1519.6)	2313.7 (722.7-5546.4)	**<0.001[b*]**
- % participants tested negative (≤ 200 mIU/ml)	15 (25.0)	3 (6.3)	**0.009[a*]**
**Immunogenicity outcome estimates**			
Anti-SARS-CoV-2 S1 RBD IgG (bau/ml)			
- Week 4	2204.6 (952.5-4406.1)	1973.9 (692.5-3428.6)	0.314[b]
- Week 12	1143.9 (488.8-2793.7)	937.9 (377.6-1736.9)	0.105[b]
- Week 24	651.4 (255.1-1482.2)	827.0 (252.0-1638.8)	0.906[b]
SARS-CoV-2 IGRA-derived IFN-γ (mIU/ml)			
- Week 12	2225.2 (1274.0-4605.0)	1622.6 (384.8-3362.7)	0.062[b]
- Week 24	1737.0 (628.1-3568.5)	2507.5 (805.3-5394.2)	0.279[b]

* p <0.05

a p-value from Chi square or Fisher exact tests, **b** p-value from Mann-Whitney tests, **c** the most recent course of rituximab treatment received by all participants was administered as follows: two doses of 1000-mg rituximab infusions separated by two weeks. Recent use was defined as rituximab-to-vaccination interval < 9 months, **d** Biologics were prescribed with the standard dosage for psoriasis. **Abbreviations:** bau, binding antibody unit; COVID-19, coronavirus disease 2019; fID, fractionated intradermal; IFN-γ, interferon gamma; IGRA, IFN-γ release assay; IQR, interquartile range; IU, international unit; ml, millilitres; NA, not applicable; SARS-CoV-2; severe acute respiratory syndrome coronavirus 2; sIM: standard intramuscular.

Table S2. Mean and 95% confidence interval of the covariates' coefficients from multivariate logistic analysis which showed statistically significant associations with the immunogenicity outcomes.

Analysis types	Outcome analysed	Covariates	Mean (95%CI)	p
ITT	Humoral immunogenicity at Week 4	Pre-intervention anti-SARS-CoV-2 IgG	0.52 (0.43, 0.60)	<0.001
	Humoral immunogenicity at Week 12	anti-SARS-CoV-2 IgG at Week 4	0.79 (0.64, 0.94)	<0.001
	Humoral immunogenicity at Week 24	anti-SARS-CoV-2 IgG at Week 12	0.80 (0.66, 0.94)	<0.001
	Cellular immunogenicity at Week 12	Pre-intervention SARS-CoV-2-specific IFN-γ response	0.41 (0.28, 0.53)	<0.001
	Cellular immunogenicity at Week 12	The doses of mRNA vaccines received prior to enrolment	-0.52 (-0.96, -0.07)	0.023
	Cellular immunogenicity at Week 24	SARS-CoV-2-specific IFN-γ response at Week 12	0.67 (0.50, 0.84)	<0.001
PP	Humoral immunogenicity at Week 4	Pre-intervention anti-SARS-CoV-2 IgG	0.60 (0.52, 0.69)	<0.001
	Humoral immunogenicity at Week 12	anti-SARS-CoV-2 IgG at Week 4	0.76 (0.58, 0.94)	<0.001
	Humoral immunogenicity at Week 24	anti-SARS-CoV-2 IgG at Week 12	0.87 (0.72, 1.02)	<0.001
	Cellular immunogenicity at Week 12	Pre-intervention SARS-CoV-2-specific IFN-γ response	0.32 (0.17, 0.47)	<0.001
	Cellular immunogenicity at Week 24	Pre-intervention SARS-CoV-2-specific IFN-γ response	0.50 (0.12, 0.88)	0.012

Abbreviations: CI, confidence interval; IFN-γ, interferon gamma; ITT, intention-to-treat; PP, per-protocol; SARS-CoV-2; severe acute respiratory syndrome coronavirus 2; sIM: standard intramuscular.

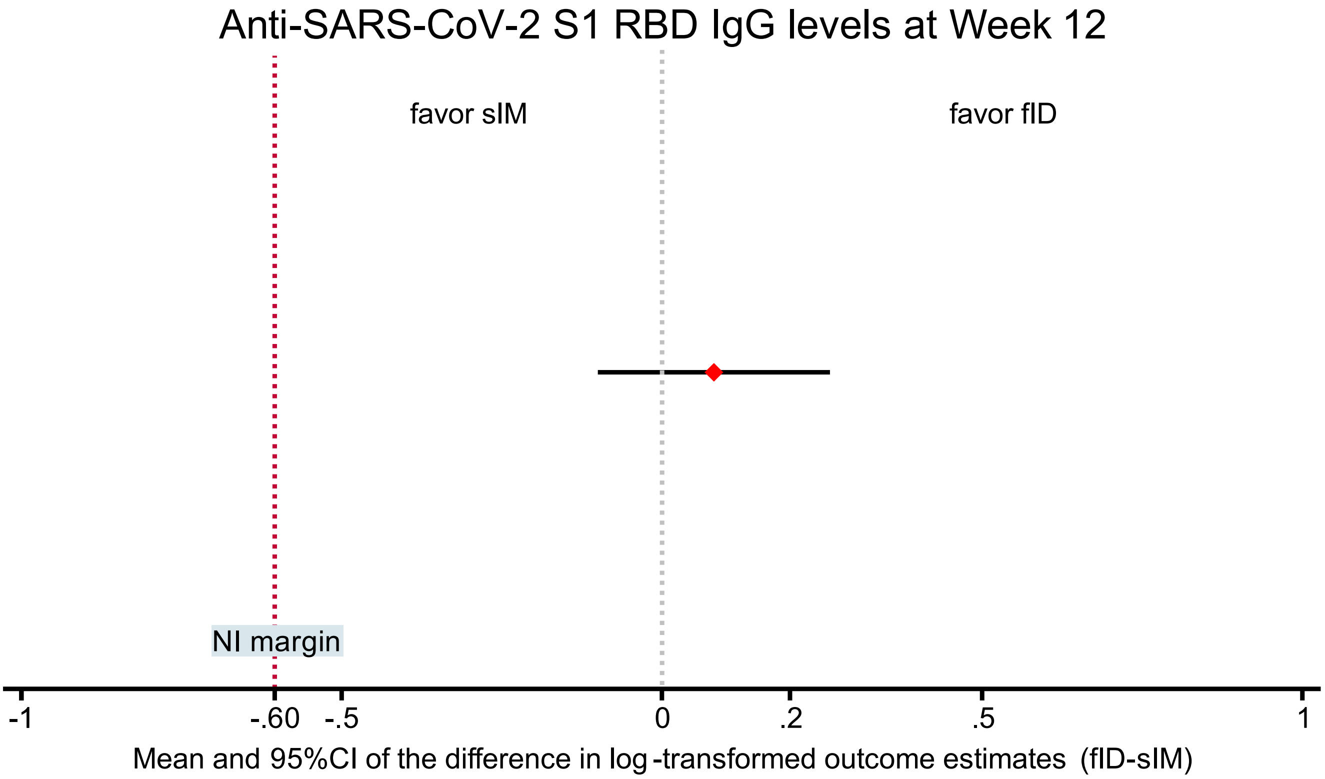

Figure S1. Intention-to-treat analysis of mean difference in the secondary humoral immunogenicity outcome estimates; anti-SARS-COV-2 IgG level 12-week post-intervention.

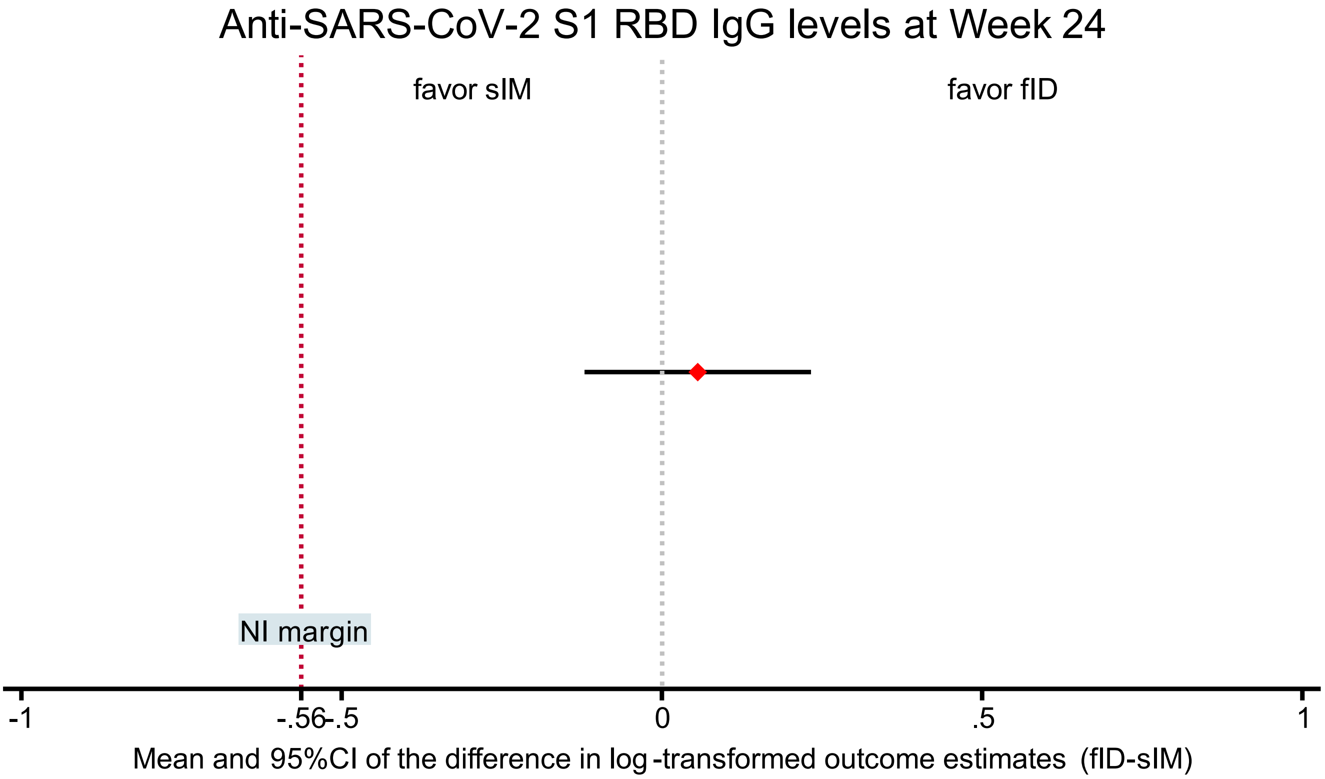

Figure S2. Intention-to-treat analysis of mean difference in the secondary humoral immunogenicity outcome estimates; anti-SARS-COV-2 IgG level 24-week post-intervention.

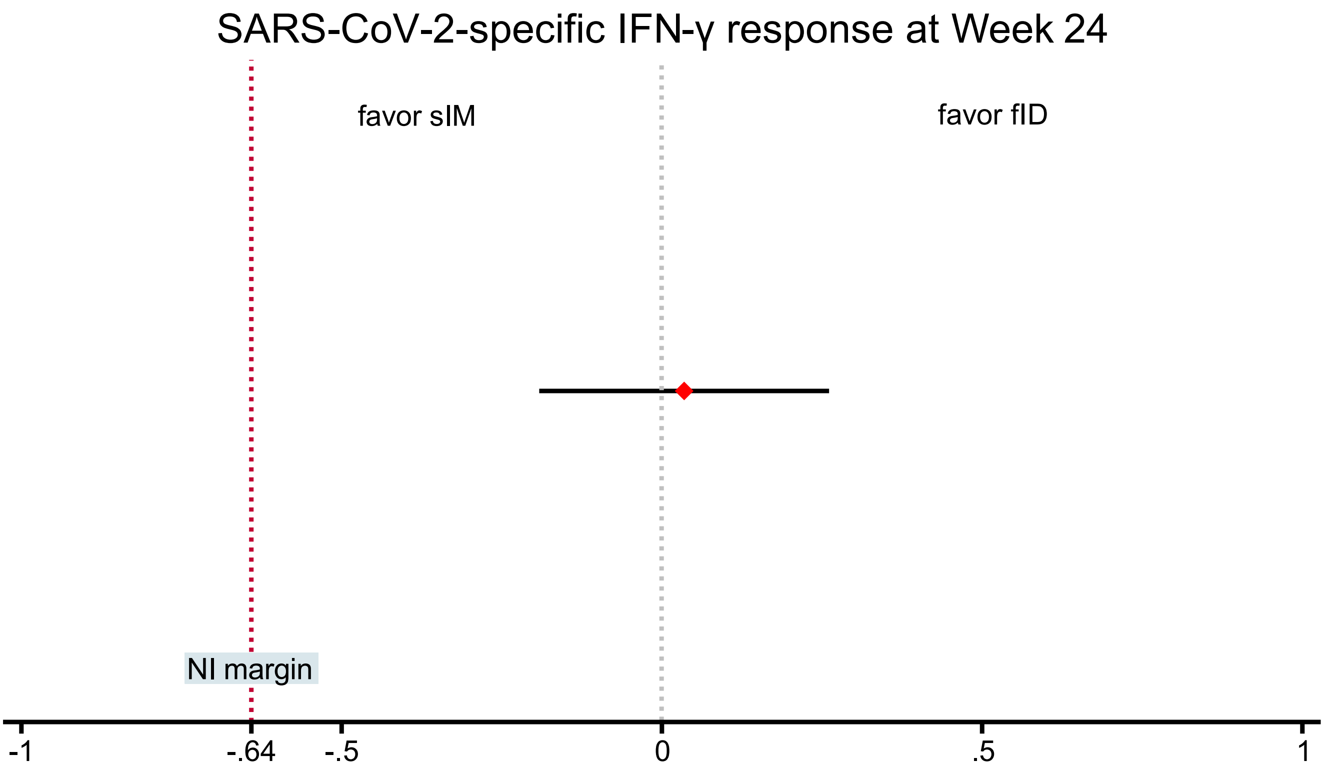

Figure S3. Intention-to-treat analysis of mean difference in the secondary cellular immunogenicity outcome estimates; IGRA-derived interferon gamma levels at Week 24.

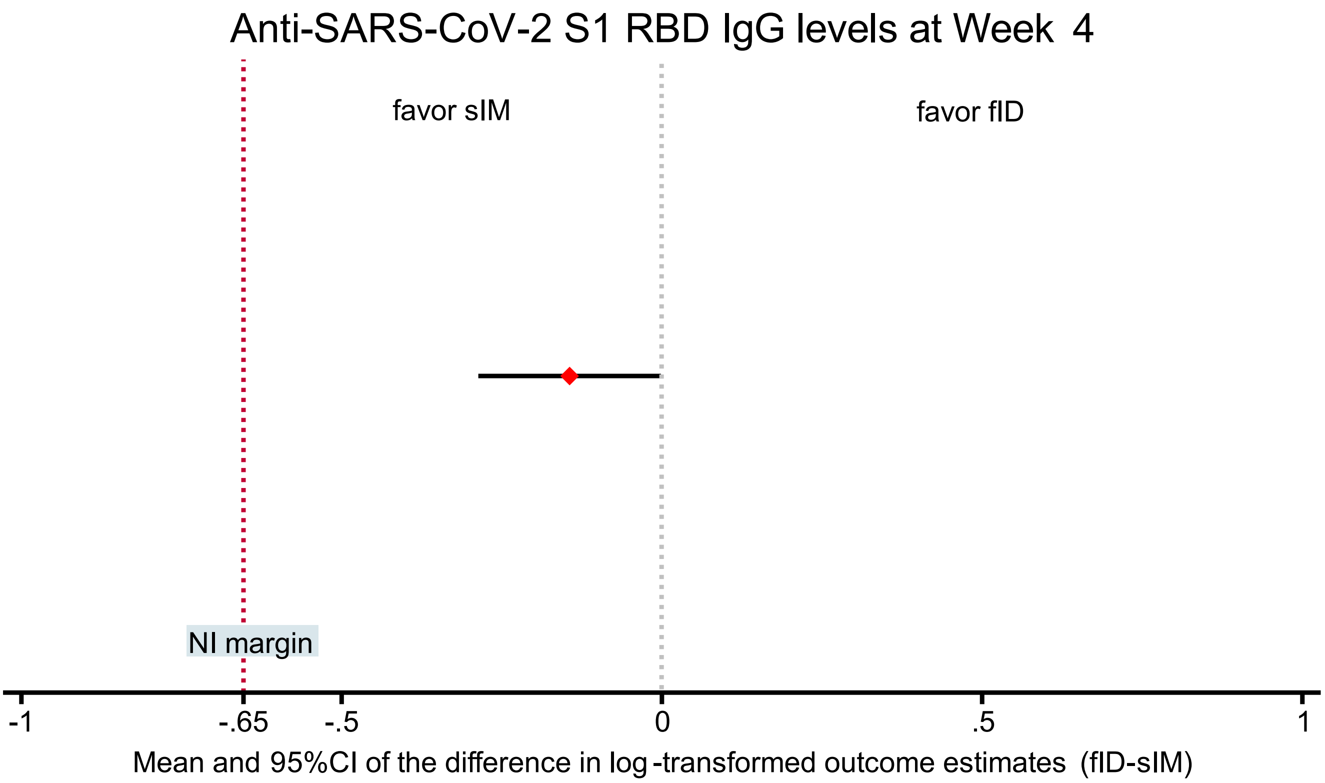

Figure S4. Per-protocol analysis of mean difference in the primary humoral immunogenicity outcome estimates.

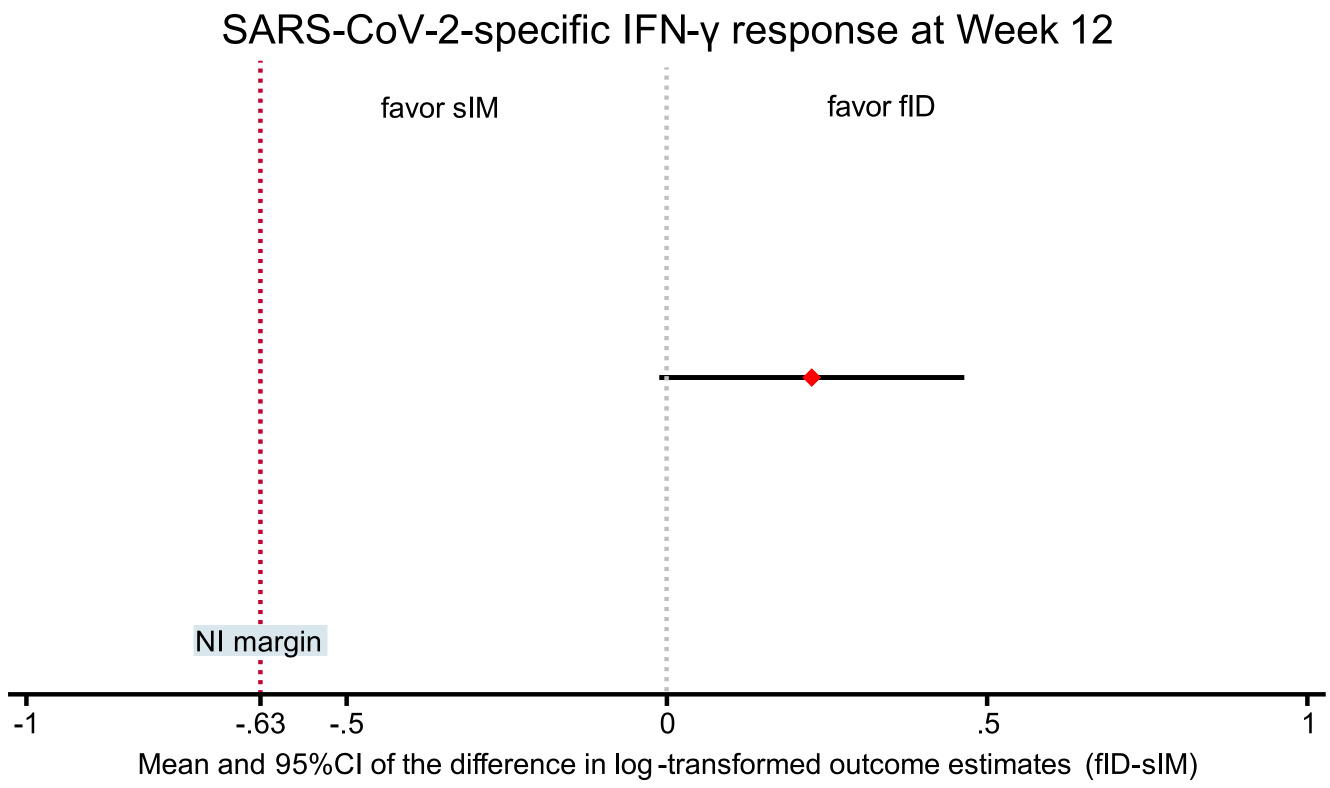

Figure S5. Per-protocol analysis of mean difference in the primary cellular immunogenicity outcome estimates.

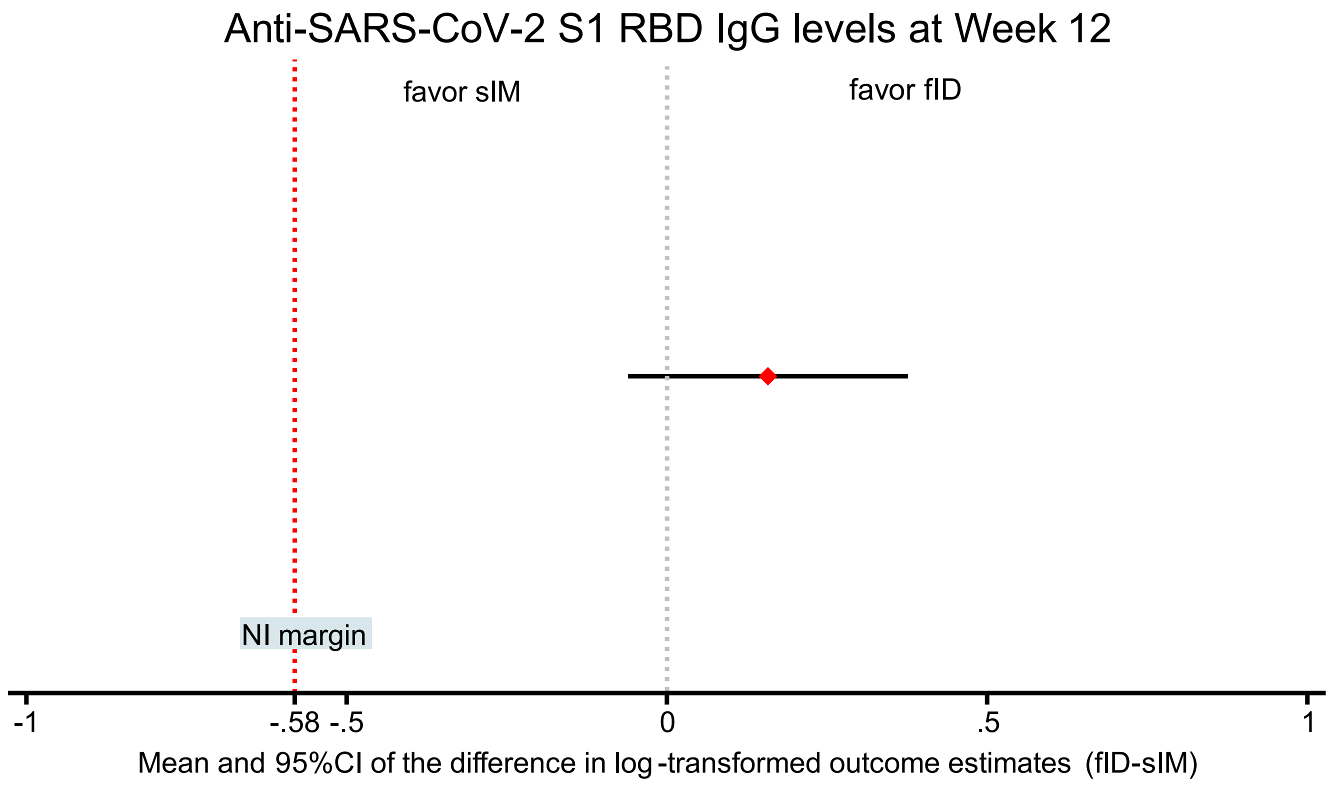

Figure S6. Per-protocol analysis of mean difference in the secondary humoral immunogenicity outcome estimates; anti-SARS-COV-2 IgG level 12-week post-intervention.

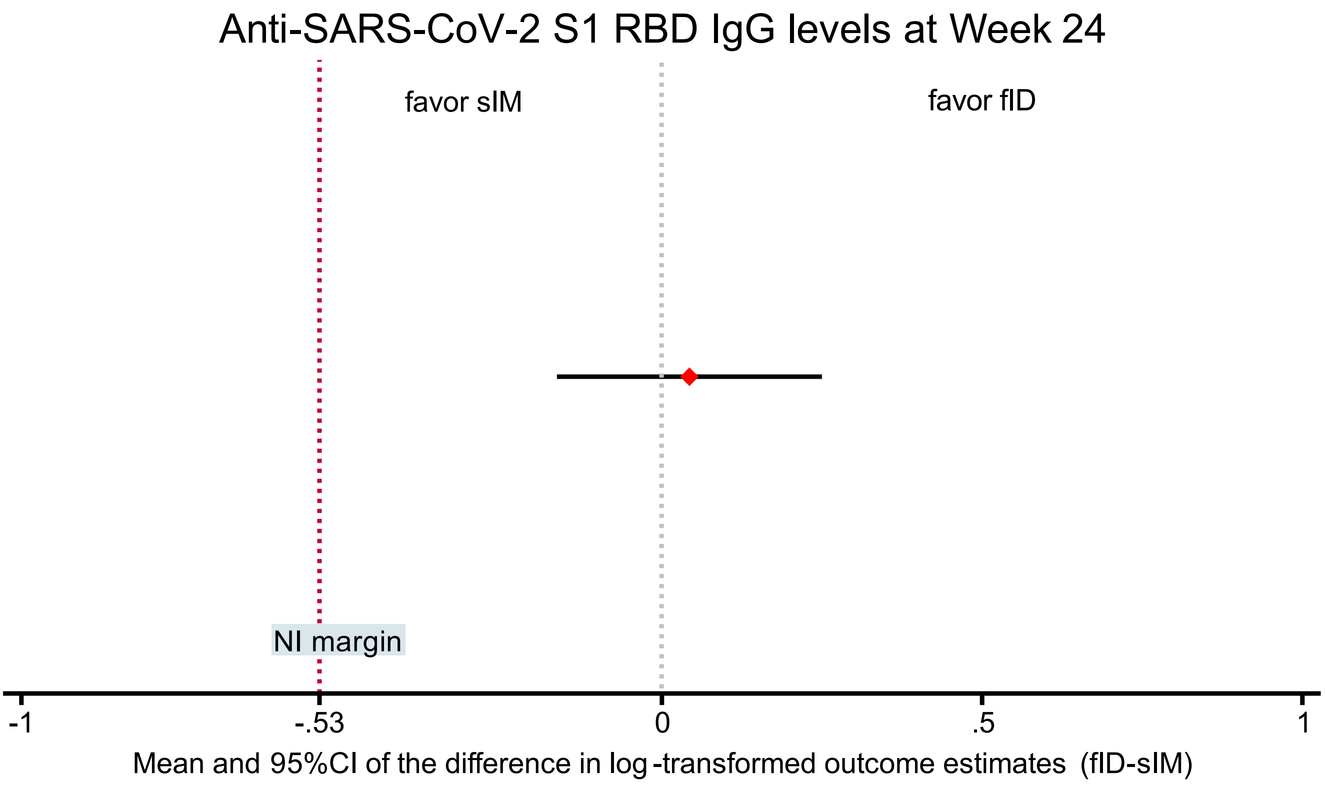

Figure S7. Per-protocol analysis of mean difference in the secondary humoral immunogenicity outcome estimates; anti-SARS-COV-2 IgG level 24-week post-intervention.

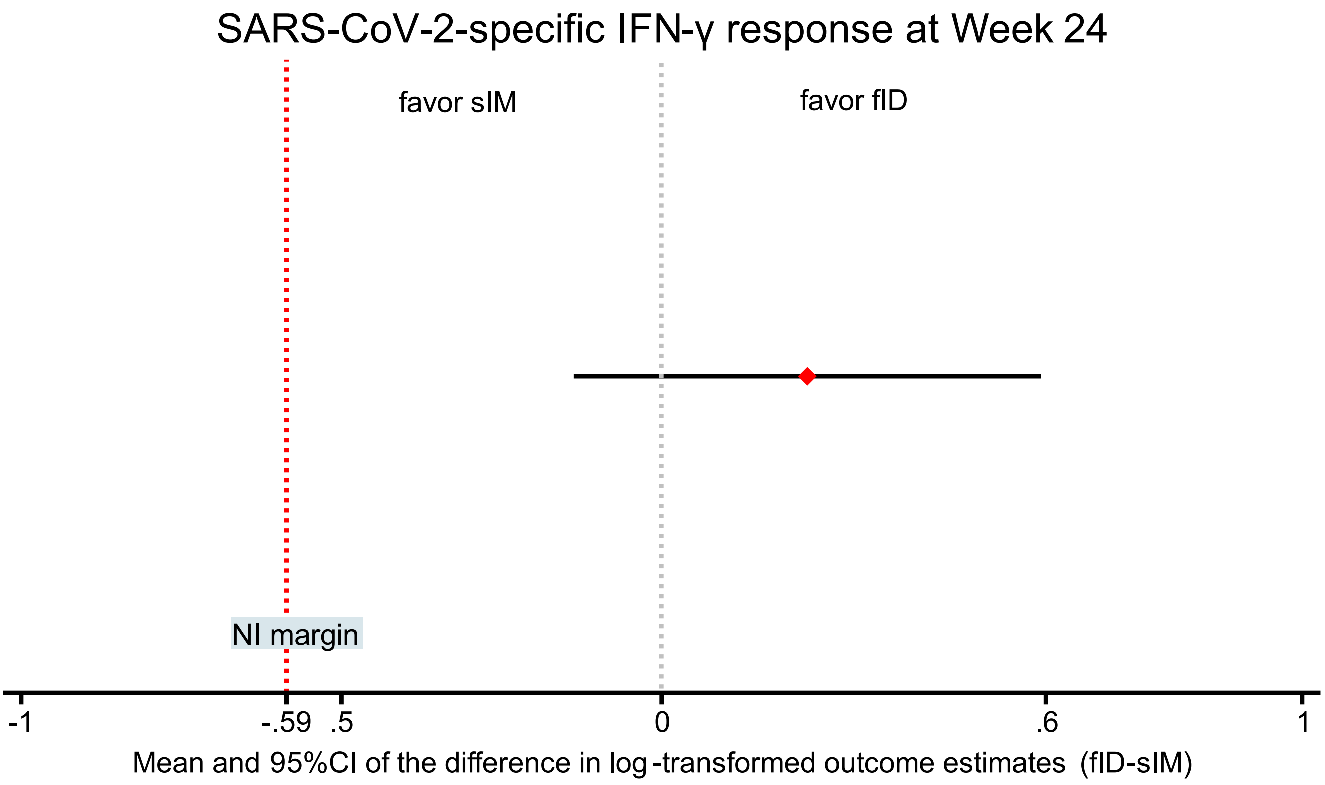

Figure S8. Per-protocol analysis of mean difference in secondary cellular immunogenicity outcome estimates; IGRA-derived interferon gamma levels at Week 24.
